# Experiences of family caregivers of patients with post-traumatic hydrocephalus from hospital to home: a qualitative study

**DOI:** 10.1186/s12913-022-08502-4

**Published:** 2022-09-07

**Authors:** Jia-nan Wang, La-mei Liu, Ronnell Dela Rosa, Meng-jie Sun, Yu-meng Qian, Meng-yao Zhuan Sun, Tong-yao Xu

**Affiliations:** 1 School of Nursing and Health, Zhengzhou University, 100 Science Avenue, High-tech district, Zhengzhou City, 450000 Henan province China; 2 School of Nursing, Philippine Women’s University, 1743 Taft Avenue, 1004 Malate, Manila Philippines; 3 Bataan Peninsula State University, College of Nursing and Midwifery, City of Balanga, 2100 Bataan, Philippines

**Keywords:** Post-traumatic hydrocephalus, Shunt, Transition care, Family caregiver, Interview, Qualitative

## Abstract

**Background:**

Post-traumatic hydrocephalus (PTH) is a complication of traumatic brain injury (TBI) that requires treatment and postoperative care. The shunt is one of the main treatments for PTH, which presents with dysfunction and infection. Considering brain injury, hydrocephalus shunt malfunction, and infection, family caregivers need to be responsible for caring for PTH patients, recognizing shunt malfunction and infection, and managing those patients accordingly from hospital to home. Understanding the experiences and needs of caregivers is beneficial for knowing their competency and quality of health care, ameliorating and ensuring future transition care. The study aimed to explore the feelings, experiences, and needs of family caregivers when caring for patients with TBI, PTH and shunts.

**Methods:**

This was exploratory research of a purposive sample of 12 family caregivers of adult patients with TBI, PTH and shunts in five neurosurgery departments at a general hospital in Zhengzhou, Henan Province, China, using a semi-structured interview method. Data were collected from October 2021 to March 2022 before being analyzed by content analysis methods.

**Results:**

Caregivers required professional and social knowledge and support in the areas of TBI, PTH and shunts, caregiving interventions, psychological care needs, and health insurance, just as caregivers do, but unlike other general caregivers, care for patients with TBI, PTH, and shunt is fraught with uncertainty and the need to manage shunt setting, and caregivers often experience 'complex emotional reaction' during the transitional period, where care needs and complex emotions may lead to a lack of caregiver confidence, which in turn may affect caregiving behaviors, and experiences that affect care may be mediated through caregiving confidence. The perceived availability of resources, particularly those that are still available to them when they return home, has a significant impact on participants' emotional response and sense of confidence.

**Conclusions:**

The emotional response and the impact of stressor caregivers after TBI, PTH, and shunt was important, and sometimes confidence in care appeared to be an intermediate and useful factor that needed to be considered as health professionals prepared to develop care resources on how to manage and empower patients with TBI, PTH, and shunt. Meanwhile, there may be gaps and inequities in supportive care for patients diagnosed with TBI, PTH, and shunt in China.

## Background

Post-traumatic hydrocephalus (PTH), defined as clinically symptomatic and radiologically confirmed communicating hydrocephalus, occurs subsequently in the weeks and months following significant traumatic brain injury (TBI) [1] with approximately 50 million new TBI cases worldwide each year [2], and PTH has been an increasingly common neurosurgical problem involving all age groups with varying series, presentations, and treatments [3–5], however, it may be difficult to identify PTH symptoms due to heterogeneity of the clinical syndromes and significant delay in presentation [6], and the epidemiology of PTH has been reported to vary widely: the estimated incidence of post-traumatic hydrocephalus (PTH) after the acute phase ranges from 12–50% [7–9]. A permanent cerebrospinal fluid shunt is the main approach to save brain function and relieve hydrocephalus symptoms [10]. A shunt is the treatment of choice for PTH, diverting excess cerebrospinal fluid from the ventricles to the abdominal cavity [11], where the peritoneum absorbs the drained cerebrospinal fluid. Several studies have reported that 5–23% of TBI patients required shunt treatment for PTH [12–14], however, the rate of PTH shunt surgery has not been collected and reported in China. In a study of 836 patients with severe TBI conducted at a large European neurosurgery center in Uppsala, Sweden, post-traumatic ventricular enlargement was found in 46% of patients, and although 3.5% (*n* = 29) of patients underwent ventriculoperitoneal shunts [11], 444 patients with severe TBI requiring long-term repair were prospectively followed by Kammersgaard et al. who found that 14.2% of patients had PTH [12]. Achieving maximum shunt survival remains a challenge [11, 15], and the incidence of shunt failure in adults has been reported to be typically around 20–30% [16, 17], with VPS infection being a serious postoperative complication with an incidence of 1.5–24.6% [18]. Patients with TBI with PTH and shunts usually require a long time for treatment.

Considering the shortened average length of hospital stay, and high hospitalization expenses under the healthcare policy, patients and their relatives choose to go home to recover after the acute stage of surgical treatment [19, 20], if there is no postoperative infection in shunt patients, the function of the shunt device was determined by clinical presentation and imaging, and patients and caregivers were usually discharged within two weeks postoperatively [16]. The transition of care from hospital to home, when patients are discharged but have not yet recovered, is a critical and vulnerable period for caregivers and patients [21]. The mainstay of long-term care is the family member who often does not have the relevant expertise and caregiving skills before the time, but who handles a range of patient care tasks alone, with nursing-related tasks including daily care, medical care, medication management, symptom management, monitoring shunt settings and shunt fault identification [22]. Caregivers encounter brain injury, PTH and the impact of shunts on daily life, sometimes complications may occur, such as epilepsy, and caregivers need to have the knowledge to recognize and manage epilepsy and, more importantly, they need to master postoperative shunt complications and shunt malfunctions [23, 24], but only have access to a limited objective shunt response, and even they have to face shunt infections [11, 25]. It is not clear whether this is a symptom or in some cases a complication of TBI or shunt malfunction, such as dizziness, headache, vomiting, etc.

Previous literature focused on TBI management, and the caregiver of traumatic brain injury (TBI) patients expressed their unsuccessful attempts to fill the gap in care coordination in Australia [26], which was inconsistent with guidelines that required patient and caregiver involvement in decision making at all stages and that care should be coordinated within and between services [27]. There is very little research on PTH and shunt, especially as PTH occurs after brain injury and shunting is a permanent way of possible dysfunction and infection [28]. What caregivers know is the material and knowledge dictated to them by professionals during their hospitalization. There is no report exploring the care conditions and experiences of these caregivers. From Mohammed’s bibliometric analysis, the predominance study of hydrocephalus management existed in the medical treatment and surgical intervention [29]. PTH patients have a brain injury and the prognosis is also important for the individuals, families, and society [23], and health professionals perceive that proper transition care can avoid hospital admissions [21]. There is a need to explore the feeling and experience of care for patients with hydrocephalus shunts to obtain or improve immediate transitional care. The purpose of the study was to explore the experiences and needs of family caregivers of TBI patients with PTH and shunt.

## Methods

We used Husserl's descriptive phenomenological approach to explore caregivers' experiences of PTH and shunt and found meaning in their experiences. Phenomenology focused on revealing the meaning of subjective experiences and speaking for itself through people's voices, which is consistent with naturalism [30]. Semi-structured interviews were used to explore the meaning of the nursing experience [31].

### Setting and sample

The study was conducted in five adult neurosurgery departments in a general hospital in Zhengzhou, Henan Province, China. These departments provide healthcare education and follow-up medical consultations. The interviews were part of larger research designed to explore caregivers' feelings and experiences of care from hospital to home, as well as the experiences of health professionals providing care. Eligible caregivers were selected through purposeful sampling. Inclusion criteria of patients: (1) moderate, or severe traumatic brain injury on admission (Glasgow Coma Score scored less than 12); (2) PTH shunt surgery within 1 year after brain injury. Caregiver inclusion criteria: (1) caregivers of patients with TBI who had PTH and underwent shunt surgery from hospital to home at least one month prior to the interview, (4) age over 18 years, (5) caregivers who undertook primary care at no cost, and (6) caregivers who were willing to share with the study team. Caregivers of patients who were (1) diagnosed as terminally ill, and (2) caregivers who opted out of treatment during the survey period were excluded.

### Data collection

Demographic data were obtained through a brief questionnaire, and the semi-structured interview guide (Table 1) based on a qualitative literature review of TBI caregivers [23, 26] and theoretical support from Fisher's Information-Motivation-Behavioral Skills Model [32] was discussed within the study group, including four experts in hydrocephalus, nursing research, psychological care, and care behavior management, who were identified and consulted to formulate the interview guide.

**Table 1:** Interview topic guide

How did you feel about caregiving patients with TBI, PTH, and shunt surgery during the past month?
What were the symptoms of the patient with TBI, PTH, and shunt surgery before discharge?
How did you do to observe or deal with these symptoms?
What are the symptoms of the patient now?
What are you told about the review?
How do you plan to care for the patient after the review?
Did you have any concerns before the review?
Did you have any needs to meet?
What support or services have you got?
Did you have any concerns after the review?
How did you feel after the review?

Data were collected from October 2021 to March 2022. Our researchers involved in patient care recruited pre-discharge caregivers under the guidance of clinicians and interviewed caregivers at the one-month post-discharge review. All interviews were conducted and recorded in Chinese by an experienced, university-educated research team, five of whom were master's students in nursing who had passed the course with distinction after systematic study in the school's qualitative research program and had experience in conducting hands-on practice in qualitative research, and the guideline was piloted with two nursing staff to identify specific questions, and an expert in the field of qualitative research was invited to a nursing expert to guide and oversee the process. In addition to listening, response and repetition, further questions were used to obtain additional details [31], such as, "Could you share the details about that? ". The interviews lasted approximately 30–60 min and continued until data saturation was reached, where the researcher repeatedly heard similar descriptions, did not acquire new categories during the analysis, and began to consider the judgment of whether the study had reached data saturation, followed by a group discussion on data saturation to achieve consistency [33]. 15 family caregivers were recruited; 12 caregivers agreed to participate; 1 caregiver declined participation due to patient deterioration; 2 caregivers declined due to work-related commitments.

### Data analysis

All interviews were digitally audio-recorded and immediately transcribed verbatim. Using Graneheim's and Lundman's conventional content analysis strategies were employed to analyze text data [34]. The analysis steps are shown below: (1) data were collected, analyzed and repeatedly read the interview transcripts by two researchers (JN and MJ) who conducted face-to-face interviews with the caregivers; (2) text about caregivers' experiences was integrated into a text that was integrated into a text that considered the entire context when the text was divided into several condensed units of meaning; (3) the condensed units of meaning were abstracted and coded by two researchers(JN and MJ); (4)two researchers(JN and MJ) collected the initial codes and classified them into subcategories and categories based on differences and similarities; (5) the data associated with each category were then reviewed and examined by two other researchers(YM and TY) unfamiliar with the transcripts, who agreed on how to rank the codes; and (6) the potential meanings of the subcategories were classified into a category [35].

### Data trustworthiness

Four criteria of credibility, confirmability, dependability, and transferability by Lincoln and Guba were used to ensure trustworthiness [36]. Credibility was established via peer checking and member checking. Two of our researchers, who were unfamiliar with the transcripts, reviewed the full data for new categories related to coding excerpts as well as member checking and invited four participants to verify textual material was consistent with their experiences [37]. The entire study process was documented in as much detail as possible in order to achieve confirmability of the study results. Peers assessed the dependability of transcripts and group discussions to ensure consistent decisions were made. We established transferability by considering the age, education levels and social role of the caregivers, the selection process and characteristics of the participants (the researcher observed and examined the behaviors of the participants), and describing the data collection and analysis process as clearly as possible [36].

### Ethical considerations

Considering the practicalities of the patient, informed consent was given only to those patients who could answer the questions correctly, but all caregivers gave written informed consent to participate in the study. The study objectives were first explained to the caregivers before the interview and written informed consent was obtained from those willing to participate. In addition, permission was granted to record interviews with a tape recorder. Participant confidentiality and anonymity were guaranteed, and participants voluntarily entered the study and could withdraw from the study at any time without consequence. The time and place of the interviews were chosen in coordination with the participants, and sometimes there might be no suggestions from the caregivers about the place of the interview, and we provided a special consultation room. This study was obtained from the Ethics Committee (ZZURIB2022-18).

## Results

Twelve caregivers completed the study. The caregivers' characteristics are presented in Table 2. The respondents' ages ranged from 24 to 57 years, 58.33% were female. Three major categories emerged from the analysis of caregivers' experiences, and subcategories associated categories are presented and described in Table 3.

**Table 2:** Demographic characteristics

Number	Gender	Age	Occupation	Educational level	Kinship with patient
1	Female	24	2	Middle school	Daughter
2	Male	45	2	Primary school	Husband
3	Male	38	2	College	Son
4	Female	40	1	College	Daughter
5	Female	35	1	High school	Daughter
6	Female	55	3	Middle school	Wife
7	Male	30	2	High school	Son
8	Female	41	2	High school	Wife
9	Female	43	3	Middle school	Wife
10	Male	48	3	Primary school	Husband
11	Male	55	3	Primary school	Father
12	Female	57	2	Primary school	Mother

(1 = full-time job;2 = Part-time job;3 = No work, including unemployment, layoff, retirement)

**Table 3:** Categories and subcategories

Categories	Subcategories
Complex emotional reaction	Uncertain reaction to patient’ s prognosis
	Discontinuity between caregivers and professionals
Inadequate confidence in caregiv-ing	Feeling insufficiency in caregiving
	Struggling to live a normal life
Unmet needs	Increasing expertise in PTH and shunt
	Developing professional organization
	Financial difficulty
	Psychological support

### Complex emotional reactions

Caregivers had complex emotional reactions to the diagnosis and shunt of patients with PTH, with disease and treatment impacting them throughout their lives, and with sub-categories uncertainty in response to the patient's prognosis, and discontinuity between caregivers and professionals.

### Uncertain reaction to patient’s prognosis

Caregivers, who may be unprofessional, are aware of the importance of the brain and the instinctive fear people have of brain injury. PTH is not present at the beginning but occurs during treatment, it occurs after trauma, bleeding, and other craniofacial injuries and surgery, and this change is fraught with uncertainty. TBI patients always had symptoms of brain injury, family caregivers couldn't see the immediate effect and improvements of the shunt, the time and improvement of the recovery consumption are full of changes and they needed more than one specialized and professional scan to determine the effectiveness of the shunt. PTH and shunt patients need to rely on shunt function for a lifetime of normal survival, but shunt complications can occur at any time.

"We all know the importance of the brain, which is the "commander-in-chief" of the human body. He had brain damage, hydrocephalus and shunt, which is terrible! "(C9)

"It was hard for me to identify something wrong that happened as a non-specialist (family caregiver), I did not know how to care for him at the beginning, but I had to take care of his life in a flurry, my own life and our family, especially his condition could change at any time. "(C4)

"The doctor said we could leave the hospital, but I didn't see any significant changes after the shunt surgery except a CT scan, which was consistent with this review results."(C9)

### Discontinuity between caregivers and professionals

Caregivers wanted to keep in touch with professionals, as they felt that physicians did not usually share too much information and engage in in-depth discussions about the patients’ recovery and needs. This resulted in caregivers not knowing the care for their patients, and to some extent, caregivers may be the dominant force, for example, they needed to pay attention to the changes in patients, including patient shunt malfunction, treatment outcomes, and at the same time, they feel discontinuity between interactions.

"We took the CT scans on time, and hoped to see the improvement compared with the last check, he(doctor) just said a few words shortly."(C9)

"He(doctor) was busy, I just had a few minutes to ask him, and it seemed that he did not want to talk more about the condition, maybe it was too obscure to understand, I didn’t know how to get better."(C12)

### Inadequate confidence in caregiving

The lack of expertise in TBI and PTH, coupled with the caregiver's perception of the severity of the patient's condition, treatment, and symptoms, resulted in a lack of caregiving capacity and efficiency. Relevant subcategories were feeling insufficiency in caregiving and struggling to live a normal life.

### Feeling insufficiency in caregiving

Family caregivers are able to realize that they are critical in monitoring the patient's condition, whether it is treatment effectiveness, recovery, or identification of shunt failures, but the fact is that most caregivers do not have the relevant expertise and experience. They are willing to do more and better to help patients recover, but there is a gap between what they actually do and what they think. This leaves them feeling inadequate in their care, especially those who care alone.

"I was still concerned about his body, stomach tube… I feared something incorrect happened in position change and feeding, which caused the condition worse. "(C11)

"I was afraid I wouldn't do enough to delay his recovery, you know, I didn't know anything about this before, but I can try everything that was a benefit for his recovery."(C3)

"During my hospital stay, when I was unsure, I could consult and confirm with my doctor or nurse so I didn't wonder if I was doing the right or wrong thing." (C1)

### Struggling to live a normal life

Caregivers reflected on the changes and challenges in their life after PTH and shunt. They saw nothing wrong with going home, but reality slapped them in the face. There were still existing symptoms of brain injury and hydrocephalus, and they needed to adjust to the presence of the shunt in all aspects of their daily lives, in addition to the possibility of shunt malfunction or infection. They had to struggle to return to normal life.

"You could not see and touch it but couldn’t ignore it, it maybe happened wrong, he is mentally exhausted, tired, and forgetful. I tried my best to get back to life like before. it was hard. I need to work, and care for a patient and our parents. I was a mother, worker, daughter and wife, every role meant one duty, everything was difficult."(C8)

"He still felt dizzy and had a headache after the operation, and his cognitive disorder persisted. Because I was worried about injuries and falls and then shunt malfunction, I would cut back on activities and give him a soft pillow at bedtime." (C6)

"She could go to the bathroom on her own during the day, but at night, she was incontinent and the symptoms of traumatic brain injury and hydrocephalus remained." (C2)

"Because she needs care and there is no one else to care for her except me for caregiving, I can’t even go out for work, not to mention the offer to socialize with friends."(C10)

### Unmet needs

Participants reported they had limited and insufficient support for caregiving TBI patients with PTH and shunt from hospital to home. The needs were diverse, with four subcategories as an example: increasing expertise in PTH and shunt, developing professional organization, financial support, and psychological adjustment.

### Increasing expertise in PTH and shunt

For patients and their caregivers, diagnosis and shunts made it hard to know about their condition. Most patients were puzzled about hydrocephalus and shunts. Improving expertise in PTH and shunts includes an understanding of hydrocephalus, as well as knowledge of shunt settings and shunt malfunctions. However, it was usually difficult to understand complex medical terminology. Here are some examples of trying to understand hydrocephalus:

"I couldn't understand the word 'hydrocephalus', why it still happened after surgery. The doctor just told me it is dangerous for his condition. We had to perform the shunt operation first. But I want to know why and what’s happened. "(C12)

"I know a little information about hydrocephalus. Due to a malfunction in the path of the injured brain, the doctor said it is caused by too much water staying in the brain, the water flows into the head and leaves away normally. His example was simple and vivid, it left me in a deep impression."(C4)

"You know what the doctor said is not enough and they are busy, we want to know more and get some help anytime. Although the Internet is convenient and accessible, I searched it on the Internet, there are lots of statements."(C1)

"In many cases, I'm not sure if it's a symptom of the injury or shunt malfunction, like if he's dizzy all the time."(C3)

### Developing professional organization

Most caregivers living in rural areas preferred to make an appointment at a local county hospital, and some of them chose to have a call with their supervising doctor. They also needed official organization source support to get reliable information and contact professional person outside of the hospital or get other help in a transitional period.

"The doctor was very responsible. When we were not sure about some problems, I usually called him for consulting, and he gave us some useful advice. We also learnt a lot from his interflow. "(C1)

"We have to consider the reliability of the information, although we can find a lot of information on the Internet." (C12)

Caregivers wanted immediate help and support when the patients presented acute illness symptoms. Their caregivers found it difficult to get continuous dynamic help to uninterruptedly meet hydrocephalus patients’ needs especially when they wanted to receive better treatment, and the process was long and volatile.

"Sometimes we went to different hospitals for more treatment, we needed to rely on our own resources to find experts and then say our complete medical treatment procedures and examination reports. It's too much work. "(C2)

"When the doctor asked us about shunt pressure, but I didn't remember it, I know that her shunt equipment of five regulations, her treatment had been given for a long time in the hospital, and the shunt pressure was changed several times. The doctor will find equipment for pressure measurement again. "(C10)

### Financial difficulty

Most adult patients with hydrocephalus had the primary disease, were mostly young and middle-aged, and were the main source of family income. On the other hand, the health insurance system had limited reimbursement and no financial and medical support policies for patients with hydrocephalus and shunt.

"His condition is serious and rehabilitation is very important to him. After we were discharged from the hospital, we kept going back and forth for specialized rehabilitation, acupuncture, and massage, but it was a long time process. The spending was expensive and had limited reimbursement from medical insurance. We couldn't afford it, so we had to do that by ourselves at home. "(C12)

### Psychological support

Constant caregiving took caregivers away from themselves and their own lives, and caregiving was seen as one's sole responsibility and lifetime, especially when traditional morals that valued filial piety above all else and focused on blood ties make it easy for caregivers to become trapped in a mental prison and require psychological adjustment.

"I had been taking care of her for a long time. Sometimes I felt that there was no hope in life. The sky seemed to be dark all the time, but I couldn't deal with it. It's deserved, if I didn't take care of her, who would take care of her? "(C6)

"Since he got sick, the focus of my life was taking care of him, I did not go out, did not work, did not play with friends, my friends also knew that there was a patient at home, and they were sorry to disturb me, even if we happened to meet I would not say anything about taking care of the patient, I felt embarrassed, and I thought it is useless to talk to them except disturbing their normal life. "(C8)

"It was important to see him as a child, not a patient. Sometimes I couldn’t control myself worried and annoyed, you just knew it was unavoidable but you could not let it occupy your whole life, change your mind. "(C7)

## Discussion

Recovery from TBI and PTH is a complex and chronic condition [38]. Although the Chinese healthcare system has struggled to achieve continuity of care and transitional care, the mismatch between population size and resource allocation is a key barrier, and we have made great progress in the management of diabetes, hypertension, and stroke, but there is still room for improvement in TBI and PTH shunt surgery. In our study, the experience of caregivers caring for patients with PTH and hospital-to-home shunts for TBI was needed for caregivers and supporters.

Complex emotional reactions have been a key perception for caregivers, especially for these patients with TBI with PTH and shunts. Brain disorders affect patient and caregiver cognition, consciousness, motor expression, and understanding, and the prognosis is unpredictable. PTH is a possible complication of TBI that can occur during treatment, and necessary shunt surgery can alleviate but not cure hydrocephalus [10, 39]. Furthermore, shunt malfunction was intangibility and the patients with malfunction might show one or more of these, lower limbs weakness, somnolence, vomiting, or bellyache, behaviors that were common and easily overlooked [40]. This explains why most caregivers exhibit complex emotional responses when caring for TBI, PTH, and shunt. However, The complex emotional reaction was a stressor that could not be neglected [41, 42], and we cannot underestimate the changes that emotional reactions bring, and to some extent, it is a burden, but for some caregivers, it motivates them to learn more about hydrocephalus and shunts in order to alleviate uncertainty, similar to previous studies of caregivers [43, 44]. Perhaps we can explore the characteristics of caregivers behind this positive influence specifically in the future.

Low confidence in care is another important experience for caregivers and is also reflected in caregivers of patients with TBI, PTH, and shunt [45]. Confidence was related to the information, competence, and experience of the process of treatment and its outcomes in PTH patients, and some studies have shown no change or even worse in the PTH status of patients in shunt [1]. Sometimes caregivers believe that professionals can consider their knowledge, skills and knowledge, and collaboration with health professionals to improve their care [26]. Then caregivers gained confidence from the actual caregiving life since the caregiving process was a continuous learning process for both patients and caregivers who improve their self-efficacy and help them to return to normal life by integrating their knowledge, experience and improving the patient's condition, which also has various effects in their actual life [46].

Confidence in caregiving appeared to be an intermediate factor in our findings that was influenced in many ways by the patient's condition, knowledge, experience, and abilities, but also by the patient's own ability to care and care behaviors. This seems to be similar to the self-care confidence in Ercole's study. Self-care confidence mediates the relationship between simple attention and self-care as well as working memory and self-care [47]. This is also supported by our qualitative study of nursing experience and nursing confidence, where factors influencing care may be realized through nursing confidence (mediated). We can imagine that in the long run, caring for patients in a hostile environment in an effort to return to a normal life brings exhaustion and low confidence, but when patients experience slight changes under care, which is feedback to the caregiver's efforts to achieve the outcome of their efforts, they receive a positive emotional response and gain more confidence, which then, in turn, influences caregiving. By even simply considering the caregiver as an individual and caregiving as a job, rather than considering role identity, we can then better understand this finding by considering confidence as a component of competence, which according to McClelland's iceberg model is in the middle of the iceberg model and can change with appropriate training or experience and then play a key role [48]. This may be an important finding for those developing resources for caregivers of patients with TBI, PTH, and shunt.

Caregivers cited unmet needs related to long-term conditions, these included expertise support, organizational support, psychological and financial support. To avoid wasting too much time in the search process, these groups need an official and mobile platform that provides reliable professional information and treatment resources. Developed countries had their official institutions and support websites dedicated to hydrocephalus, which was very friendly for hydrocephalus patients and their caregivers [49], while we can consider the advantages of peer support in the Chinese cultural context to realize such shunt patients and caregivers to share experiences and learning, which may bridge the gap between self-care and professional help and promote a positive reconstruction of postoperative life [50, 51]. Considering the limited medical resources and insurance system, some expensive surgical equipment and medications are not available in China with only self-pay reimbursement, especially for neurosurgical patients, and some improvements may need to be considered for future hydrocephalus equipment facilities and healthcare systems.

### Strengths and limitations

This study described the experiences and needs of family caregivers with PTH and shunt. As a qualitative study, there were a few limitations, first, we selected a sample of caregiving patients with TBI, PTH, and shunt during an approximately one-month transition from hospital to home in a large general hospital who demonstrated that their experiences with patients with PTH shunt were unique and complex, and not well, due to geographic constraints, the caregivers we interviewed were from both urban and rural areas of the city where the hospital was located. In China, there are differences in the availability of healthcare resources in these areas (e.g., cities, towns, and rural areas), which can greatly impact the caregiver experience. The participants we interviewed were located in the most populous regions of China, and other caregivers in different regions may have different experiences in providing care to patients with TBI with PTH and shunts. Second, we recruited caregivers before patients were discharged from the hospital and interviewed caregivers one month after discharge. This is the information we obtained. However, when TBI patients with PTH and shunts were discharged with the same instructions and education, we could not avoid the presence of patients who did not come to the hospital one month later. The reasons behind this phenomenon need to be further explored.

## Conclusion

This study interviewed family caregivers about their experiences and needs in caring for patients with PTH and shunts one month after discharge home from the hospital. Patients, caregivers, and health care providers seem to underestimate the stress and impact of stressor caregivers after TBI, PTH, and shunts, most caregivers are willing to try and adapt to the new changes in our study, and caregiving confidence seems to be an intermediate and useful factor when health professionals are ready to develop how to manage and empower patients with TBI, PTH, and shunts, and these points are helpful and need to be considered. The results of this study help health professionals recognize the unique supportive care needs of caring for patients with PTH and shunts with TBI, and future research needs to explore more experiences in different aspects of caring for patients with PTH and shunts with TBI.

## Data Availability

The data that support the findings of this study are available from authors but restrictions apply to the availability of these qualitative data, which were used under license for the current study, and so are not publicly available. However, data will be available from first author (Jia-nan Wang, email: 202,022,492,017,162@gs.zzu.edu.cn) upon reasonable request.

## Abbreviations

PTH: Post-traumatic hydrocephalus
TBI: Traumatic brain injury
